# Resilience definitions, theory, and challenges: interdisciplinary perspectives

**DOI:** 10.3402/ejpt.v5.25338

**Published:** 2014-10-01

**Authors:** Steven M. Southwick, George A. Bonanno, Ann S. Masten, Catherine Panter-Brick, Rachel Yehuda

**Affiliations:** 1 Department of Psychiatry, Yale University School of Medicine, New Haven, CT, USA; 2 National Center for Post-Traumatic Stress Disorder (NCPTSD), VA Connecticut Healthcare System, West Haven, CT, USA; 3 Department of Counseling and Clinical Psychology, Teachers College, Colombia University, New York, NY, USA; 4 Institute of Child Development, University of Minnesota, Twin Cities, Minneapolis, MN, USA; 5 Department of Anthropology & Jackson Institute, Yale University, New Haven, CT, USA; 6 Division of Traumatic Stress Studies, Department of Psychiatry, James J. Peters Bronx VA and Ichan School of Medicine at Mount Sinai, New York, NY, USA

**Keywords:** Resilience, stress, trauma, post-traumatic stress disorder

## Abstract

In this paper, inspired by the plenary panel at the 2013 meeting of the International Society for Traumatic Stress Studies, Dr. Steven Southwick (chair) and multidisciplinary panelists Drs. George Bonanno, Ann Masten, Catherine Panter-Brick, and Rachel Yehuda tackle some of the most pressing current questions in the field of resilience research including: (1) how do we define resilience, (2) what are the most important determinants of resilience, (3) how are new technologies informing the science of resilience, and (4) what are the most effective ways to enhance resilience? These multidisciplinary experts provide insight into these difficult questions, and although each of the panelists had a slightly different definition of resilience, most of the proposed definitions included a concept of healthy, adaptive, or integrated positive functioning over the passage of time in the aftermath of adversity. The panelists agreed that resilience is a complex construct and it may be defined differently in the context of individuals, families, organizations, societies, and cultures. With regard to the determinants of resilience, there was a consensus that the empirical study of this construct needs to be approached from a multiple level of analysis perspective that includes genetic, epigenetic, developmental, demographic, cultural, economic, and social variables. The empirical study of determinates of resilience will inform efforts made at fostering resilience, with the recognition that resilience may be enhanced on numerous levels (e.g., individual, family, community, culture).

For decades, the fields of neuroscience, mental health, medicine, psychology, and sociology have been collectively focused on the short-term and long-term consequences of stress, and more recently, extreme stress. Stress is a reality of our daily lives. At some point, most people will be exposed to one (or more) potentially life-threatening traumatic experiences that can influence mental health and result in conditions such as post-traumatic stress disorder (PTSD) (Karam et al., 2014). These severe adversities include exposure to interpersonal violence, the trauma of war, death of a loved one, natural disasters, serious industrial or other accidents, and terrorism (American Psychological Association, 2010; Dimitry, 2012; Eisenberg & Silver, 2011; Furr, Comer, Edmunds, & Kendall, 2010; Masten & Narayan, 2012; Masten & Osofsky, 2010; Norris, Tracy, & Galea, 2009; Osofsky & Osofsky, 2013; Tol, Song, & Jordans, 2013). Some stressors are ongoing, such as the stress of exposure to bullying, harassing work-place environments, dysfunctional or challenging relationships, the grinding stress of poverty, and even the impact of environmental stressors such as extreme weather conditions and global warming (Arnold, Mearns, Oshima, & Prasad, 2014; Evans, Li, & Whipple, 2013; Lundberg & Wuermli, 2012). When stress exposure is unusually intense, chronic, uncontrollable, and overwhelming, it can give rise to—or exacerbate—burnout, depression, anxiety, and numerous physical conditions, such as inflammatory, cardiovascular, or other medical illnesses (Karatoreos & McEwen, 2013; Russo, Murrough, Han, Charney, & Nestler, 2012; Southwick & Charney, 2012a, 2012b; Southwick, Litz, Charney, & Friedman, 2011; Southwick, Vythilingam, & Charney, 2005).

Yet, just as there is concern about the deleterious effects of trauma exposure, there is also unprecedented interest in resilience. This paper summarizes key points that emerged as the topic of resilience was discussed from a comprehensive, interdisciplinary perspective during the opening plenary meeting of the 29th Annual International Society for Traumatic Stress, held in Philadelphia, Pennsylvania, in November, 2013. The discussion was chaired by Steven Southwick, M.D. Panelists included: Ann Masten, Ph.D., George Bonanno, Ph.D., Catherine Panter-Brick, Ph.D., and Rachel Yehuda, Ph.D. Dr. Southwick posed a series of questions about resilience to each of the panelists.

## Panel discourse

### Dr. Steven Southwick: The evolving definitions of resilience

Most of us think of resilience as the ability to bend but not break, bounce back, and perhaps even grow in the face of adverse life experiences. The American Psychological Association (2014) defines resilience as “the process of adapting well in the face of adversity, trauma, tragedy, threats or even significant sources of stress (para. 4).” While this definition is useful, it does not reflect the complex nature of resilience (see Southwick, Douglas-Palumberi, & Pietrzak, 2014 for a discussion). Determinants of resilience include a host of biological, psychological, social and cultural factors that interact with one another to determine how one responds to stressful experiences.

In defining resilience, it is important to specify whether resilience is being viewed as a trait, a process, or an outcome, and it is often tempting to take a binary approach in considering whether resilience is present or absent. However, in reality, resilience more likely exists on a continuum that may be present to differing degrees across multiple domains of life (Pietrzak & Southwick, 2011). An individual who adapts well to stress in a workplace or in an academic setting, may fail to adapt well in their personal life or in their relationships.

Resilience may change over time as a function of development and one's interaction with the environment (e.g., Kim-Cohen & Turkewitz, 2012). For example, a high degree of maternal care and protection may be resilience-enhancing during infancy, but may interfere with individuation during adolescence or young adulthood. In addition, our response to stress and trauma takes place in the context of interactions with other human beings, available resources, specific cultures and religions, organizations, communities and societies (see Sherrieb, Norris, & Galea, 2010; Walsh, 2006). Each of these contexts may be more or less resilient in their own right and. therefore, more or less capable of supporting the individual.

The more we can learn about resilience, the more potential there is for integrating salient concepts of resilience into relevant fields of medicine, mental health and science. This integration is beginning to foster an important and much needed paradigm shift. Rather than spending the vast majority of their time and energy examining the negative consequences of trauma, clinicians and researchers can learn to simultaneously evaluate and teach methods to enhance resilience. Such an approach moves the field away from a purely deficit-based model of mental health, toward the inclusion of strength and competence-based models that focus on prevention and building strengths in addition to addressing psychopathology.

In the following section, four scientists from different disciplines reflect on how their understanding and definition of resilience has evolved in the course of their research.

### Dr. George Bonanno: Resilience as a stable trajectory of healthy functioning

In our research, we are interested in following people over time. We define resilience very simply as a stable trajectory of healthy functioning after a highly adverse event. Our work (e.g., Bonanno, 2004; Bonanno, Westphal, & Mancini, 2011) has typically focused on acute life events, what we call potentially traumatic events. Over the course of time, often for a number of years, we map out the trajectories of people's responses to those events (e.g., Bonanno, Kennedy, Galatzer-Levy, Lude, & Elfstrom, 2012; Bonanno et al., 2012; DeRoon-Cassini, Mancini, Rusch, & Bonanno, 2010; Orcutt, Bonanno, Hannan, & Miron, 2014). What we call a resilience trajectory is characterized by a relatively brief period of disequilibrium, but otherwise continued health (Bonanno, 2004; Bonanno et al., 2011). In our research we have found that the resilience trajectory is very common, that it is not simply the absence of psychopathology, and that it is distinct from other patterns of response to potentially traumatic events, some of which are neither pathological nor resilient (Bonanno, 2004, 2012; Bonanno, Brewin, Kaniasty, & LaGreca, 2010; Bonanno et al., 2011).

Dr. Southwick talked about a paradigm shift where we have begun to ask questions that really haven't been asked before, such as why are most people able to cope so well?

In addition to focusing on what goes wrong with people who become chronically symptomatic and function poorly after adversity, we have begun to ask about what goes right in people who negotiate potentially traumatic events with equanimity. What are the natural mechanisms that allow most people to cope successfully with adversity? What are they doing and how are they coping?

Over the past 20 years I have come to value the importance of focusing on what we can understand empirically. It is extremely important to develop operational definitions for resilience because resilience, like trauma, is one of those words that has colloquial meaning. During general discourse, people talk about trauma and resilience in very loose terms. But when trauma researchers talk about trauma they have specific definitions in mind. And as a field we struggle with these definitions. I see the same issue with resilience, particularly since there is currently so much interest in building resilience. But we need to keep our focus on empirical data in order to determine exactly what we mean by resilience, and in order to measure it reliably. That takes time and a great deal of research.

### Dr. Rachel Yehuda: Resilience may co-occur with PTSD: Moving forward in an insightful and integrated positive manner

Dr. Bonanno, I'm very intrigued by your definition of resilience, but I don't know whether the trajectory of resilience you describe allows resilience to co-occur with PTSD or other illnesses that are associated with a traumatic event. My own view is that trauma survivors who develop PTSD may be just as resilient as trauma survivors who don't develop PTSD (Yehuda & Flory, 2007). When I first started to focus in this area, like many people, I thought of resilience as the opposite of psychopathology or PTSD—that trauma survivors could be split into two groups, those who had PTSD and those who were resilient. Then we (e.g., Yehuda, Bierer, Pratchett, & Pelcovitz, 2010; Yehuda & Flory, 2007; Yehuda et al., 2013) began to make a distinction in the non-PTSD group between resistance (e.g., survivors who did not develop psychopathology) and recovery (e.g., survivors who did develop PTSD, or other symptoms, but who no longer had those symptoms). This got me thinking that resistance depicted as never developing symptoms to adversity is not the same thing at all as having symptoms and bouncing back. I have to admit that the best description of resilience is one I heard on TV, in connection with a Timex watch commercial. The watch was described as having the ability to “take a licking and keep on ticking.” So, for an inanimate object, the quality of never breaking despite exposure is a good definition, but for a person, perhaps it is better to conceptualize resilience as a process of moving forward and not returning back. When a watch is dropped, it doesn't improve. But people who are traumatized sometimes do actually end up in a better place than they started in many respects. In light of that, my current definition of resilience as it applies to people would involve a reintegration of self that includes a conscious effort to move forward in an insightful integrated positive manner as a result of lessons learned from an adverse experience. The idea of moving forward is an important component of resilience for me because this notion recognizes that some of the most resilient people, at least that I know, may have had or still have very severe PTSD that they struggle with every day. But they don't succumb to its negative effects. To me, resilience involves an active decision, like sobriety, that must be frequently reconfirmed. That decision is to keep moving forward.

### Dr. Ann Masten: Resilience as the capacity of a dynamic system to adapt successfully

I started work in this area when I went to the University of Minnesota. I was recruited there by Norman Garmezy, who was one of the pioneers in the study of resilience in children (Masten, 2014a, 2014b; Masten & Cicchetti, 2012). As Dr. Bonanno was saying, I think my views have been influenced by the nature of our work. Over the years, I have studied normative populations of school children, as well as homeless families and young survivors of war and other severe adversities (Masten, 2014b; Masten & Tellegen, 2012; Masten et al., in press). As you know, there is considerable research now on adverse child experiences across the country, and often people are surprised by the frequency with which adults report that they experienced all kinds of traumatic events during childhood (Center for Disease Control and Prevention, 2013; Felitti et al., 1998). We see this in our prospective longitudinal research with community samples of families (Masten, 2014b; Masten & Tellegen, 2012). Minnesota is a refugee destination, where we have had an influx of Cambodian men and women, as well as many other war refugees from around the world. In recent years, we have seen a great many refugees from Eastern Europe and African countries. Observing their trauma symptoms and recovery has influenced my thoughts about resilience (see Masten, 2014b). The Cambodian refugees who came to Minnesota as young people were children when they were exposed to the horrors of the Khmer Rouge regime. In Minnesota, we have one of the largest, if not the largest, concentration of survivors in the United States from that tragic period. Many of these young people would have periodic flare ups of PTSD symptoms. To echo what Dr. Yehuda said, they did not leave their traumatic experiences and symptoms behind, yet I believe everyone would describe them as a remarkably resilient group of people. These were true survivors. When people asked us if we had a comparison group, we could find only one person in our region of the United States who represented a reasonable comparison, because he left Cambodia just before the killings began. That individual was a foot taller than any other person in our sample, likely due to the advantages in nutrition and protein intake he experienced. In other words, the comparison group did not make it out of the “killing fields.” The Minnesota refugees were all survivors, and many of these young people were getting on with their lives.

These young Cambodians certainly varied in how well they were functioning in different domains of their life at school or work and at home. As Dr. Southwick noted, multiple domains of life need to be considered in thinking about resilience, and individuals usually vary across domains in how well they are functioning.

For many years I've also studied other common adversities that children and adolescents face—not only in Minnesota, but all over the world, including poverty, mobility, homelessness, and migration (see Masten, 2014b; Masten, Liebkind, & Hernandez, 2012; Masten et al., in press). These experiences have influenced how I think about resilience. I have also been influenced by interacting with professionals in other fields who are concerned with resilience. Over the past four or five decades, the notion of resilience has been taken up by many different disciplines. If you are interested in understanding the impact of major traumatic events like natural disasters, industrial disasters, global climate change, terrorist attacks, and war on child development, you have to think in terms of multiple interacting systems. Sitting down at the table with people who study engineering resilience, resilience in ecologies, resilience in communities and so forth has profoundly swayed my thinking. Over the years, the definition of resilience in my work has become much more systems oriented (Masten, 2014a, 2014b; Masten & Monn, in press). I am looking for a broad conceptual definition of resilience that is scalable across different disciplines and levels of analysis.

Currently, my favorite definition is that resilience refers to the capacity of a dynamic system to adapt successfully to disturbances that threaten the viability, the function, or the development of that system (Masten 2014a, 2014b). I think this kind of definition facilitates the ability to think through and work together with people who are trying to prepare populations for dealing with disasters. We want to build that kind of capacity to adapt. I think it is also the kind of definition you can use across system levels, from a molecular level to the levels of human behavior in family, community or even societal contexts. You can also talk about resilience in economies and so forth.

There are many issues we have to deal with when we take a broad definition like this one. As a scientist, I have to define what I mean by “capacity.” What does it mean to adapt successfully? I want to be able to measure it. As a developmental scientist, I'm also interested in how well children are doing in all of the age-salient developmental tasks that we expect children to achieve as they move along in life. But, of course, those kinds of developmental tasks are going to vary historically, culturally, and even geographically. I'll hold off on the rest of my comments and turn it over to the anthropologist.

### Dr. Catherine Panter-Brick: Resilience as a process to harness resources to sustain well-being

I am interested in the important issue of how resilience is understood across different cultures. How do we construct culturally relevant definitions of resilience? I study risk, resilience, and health in settings of violence and poverty (Panter-Brick, 2014). For instance, I have studied resilience to famine in Niger, among homeless street-children in Nepal, and in the wake of war in Afghanistan (e.g. Panter-Brick, Goodman, Tol, & Eggerman, 2011; Panter-Brick, Grimon & Eggerman, 2014; Panter-Brick, Grimon, Kalin, & Eggerman, in press). I work with humanitarian organizations to articulate what it means to promote resilience and develop resilience-building interventions in challenging settings. While humanitarian organizations very much appreciate the rhetoric of resilience, they experience some frustrations with the “toolkit” available to measure and evaluate it.

So I agree with the idea of keeping it simple, but let us avoid what I call the three “deadly sins of resilience research.” One of these deadly sins is to be conceptually hazy with respect to how we articulate resilience in settings that are different from our own. A second deadly sin is to be empirically light with respect to actively seeking evidence on resilience in a broad range of contexts—for children and adults, veterans and civilians, western and non-western societies. And the third sin is to be methodologically lame with respect to how we measure resilience, especially in places where cultural goals and cultural resources are less familiar to us. When we are conceptually hazy, empirically light, and methodologically lame, we fall prey to three deadly sins in resilience research (Panter-Brick & Leckman, 2013).

To my mind, resilience is a process to harness resources to sustain well-being (Panter-Brick & Leckman, 2013). I like the word “process” because it implies that resilience is not just an attribute or even a capacity. I like the phrase “to harness resources” because it asks us to identify what are the most relevant resources to people in places like Afghanistan, Niger, or the United States. And I like the expression “sustained well-being” because resilience involves more than just a narrow definition of health or the absence of pathology. So I would define resilience as “a process to harness resources in order to sustain well-being.”

### Dr. Steven Southwick: Determinants of resilience

It sounds like all of you over time have changed your definitions of resilience based on research and your own experiences.

What about the determinants of resilience? What makes some people more resilient than others? What have we learned from resilience science? From your area of expertise and from your perspective, what are the most important determinants or drivers of resilience?

### Dr. Rachel Yehuda: Biological underpinnings

I don't really know what makes some people more resilient than others. If we think about resilience as a stable trajectory or predictive trait, then we can think about biological underpinnings or even one's genes as important determinants (Simeon et al., 2007; Yehuda, Flory, Southwick & Charney, 2006; Yehuda et al., 2013). However, when we think about resilience as a process, then we are talking about an organism that is actively interacting with an environment. This does not rule out biological or even genetic contributors, but it might modify our understanding of how environmental events contribute to biological changes, rather than the other way around. I would imagine that what makes some people more resilient than others would be better support systems, better opportunities, better DNA, and a host of other non-DNA factors either appearing alone or interacting with one another. There are many different factors that could make some people more resilient than others. But the prominence of a biological underpinning of resilience is going to depend on our definition of whether resilience is a trait that determines a response to adversity or results from environmental engagement.

A very simple way to begin to address this issue is to do longitudinal studies. In our laboratory, we have been studying how biological variables that are measured after trauma exposure change in people who are treated for PTSD. As we all know, some people respond better than others, and many do not respond to specialized PTSD psychotherapy. By asking about biological changes before and after treatment in responders and non-responders to treatment, it is possible to know whether responders are different biologically even before treatment is administered. That would suggest that predictors of recovery are predetermined even before treatment begins. However, if responders and non-responders only differ from each other biologically at post-treatment, this would indicate that what actually happens in treatment is the critical determinant, and that biological correlates of recovery can occur in anyone who responds to a therapeutic modality.

We like to think that response to therapy depends on the type of therapeutic approach or the skill of the therapist. However, this simple paradigm can actually tell us whether variations in biology predict who will or who will not respond successfully to therapy (Yehuda et al., 2010, 2013). We could also expect some biological changes to associate with recovery since biology changes and adapts to the environment and is highly influenced by numerous other factors, such as available resources and your own internal drive to fight. The decision to fight back against adversity is a complicated one that many people have the remarkable capacity to make.

### Dr. Ann Masten: Interacting systems

The capacity for resilience in humans is distributed across many interacting systems (Masten, 2014a, 2014b; Masten & Monn, in press). We are a social species. I have argued in the past (Masten, 2001) that there are fundamental adaptive systems that have come down to us through biological and cultural evolution and these are constantly being created and constantly changing. We are all living human systems that interact continuously with our environments. It's all about process. When you think of young children, for example, they are products of evolution and they are very adaptive. They have a lot of inherent adaptive capacity. But part of that capacity is embedded in the caregiver bond. I think it is very interesting to consider the adaptive systems that are common to humans as well as other closely related social species. Some systems, like the way our attachment systems work at a biological level and a behavioral level, are very similar to the way they work in other species. But we also are a species that has been influenced by cultural evolution and we have freed ourselves from biology through our capacity for language, learning and memory.

So we are able to pass down a tremendous amount of knowledge about what helps, what works and what doesn't work. Some of our capacity comes from our inherent potential and some from what we learn over time. A human brain in good working order has tremendous capacity to learn and pick up information about how to cope. Our self-regulation skills are vitally important for adapting to many kinds of threats to human experience. Much of resilience, especially in children, but also throughout the life span, is embedded in close relationships with other people. Those relationships give you a profound sense of emotional security and the feeling that someone has your back, because they do. As we get older we have the capability for spiritual relationships as well as friendships with other human living contemporaries and again we draw great capacity for adaptation from those relationships.

Another extremely important adaptive system that needs more research at many levels of analyses is the mastery motivation system. This system was identified decades ago and we see this in very young children. We get a kick out of “doing” and interacting successfully in the environment. You can easily see this when you watch young children throwing things off the highchair or walking for the first time with great delight. The mastery motivation system is a very powerful driver of learning and resilience (Masten, 2014b). As a clinician I think it's challenging to help people if that system is shut down for any reason. Mastery motivation is a powerful driver of resilience and this adaptive system is another one that you see across multiple species. Again I'm going to yield to our anthropologist to talk about the capacity that is embedded in cultures. Although there is more research in recent years on cultural aspects of resilience, cultural processes generally have been understudied. Now there is growing focus on the ways in which people all over the world draw on cultural practices, beliefs and learning and support from each other to endure and recover from all kind of challenges.

### Dr. Panter-Brick: Cultural resilience

As I hear about the biological perspectives on resilience and the developmental mastery perspective on resilience, I want to add a few words on a cultural perspective on resilience. Let me give you an example. I conducted systematic face-to-face interviews with over a thousand families, both youth and adults, in Afghanistan. If you had to boil down “resilience” to just one single word, in the Afghan context, that word is “hope.” In my work, I found that Afghan families believe that the future matters much more than the past in determining their present well-being: being able to get up each day and go harness resources toward securing a better future matters more than the turmoil and traumas of the past. For me, what makes some families more resilient than others is their ability to hang on to a sense of hope that gives meaning and order to suffering in life and helps to articulate a coherent narrative to link the future to the past and present. That hope or “meaning-making” is the essence of a cultural perspective on resilience (Panter-Brick & Eggerman, 2012). In the words of *Václac Havel*, the playwright and dissident who led the creation of the first Czech Republic, “Hope is not the conviction that something is going to turn up well, but the certainty that something makes sense, however things are going to turn out” (as cited in Eggerman & Panter-Brick, 2010). What matters to individuals facing adversity is a sense of “meaning-making”—and what matters to resilience is a sense of hope that life does indeed make sense, despite chaos, brutality, stress, worry, or despair.

A body of lots of work has also documented the social ecology of resilience, which includes studying how key resources in the social, economic, cultural, or political environment influence individual-level or family-level resilience. This work implies that, when designing interventions, the “arrow of change” can be pointed from the society to behavioral or developmental outcomes: rather than tinker with individual-level capacities to cope, we must change the society-level odds stacked against individuals that block their opportunities to achieve a better future (e.g., Reed, Fazel, Jones, Panter-Brick, & Stein, 2012). For me, that's really the essence of a cultural and social perspective on resilience. We need to provide people with the resources that facilitate their ability to create a better future and construct meaning in life. We can also constructively think of “structural resilience”—building robust structures in society that provide people with the wherewithal to make a living, secure housing, access good education and health care, and realize their human potential (Ager, Annan, & Panter-Brick, 2013).

### Dr. Steven Southwick: Focusing on what comes after trauma and not the trauma itself

This is in line with the philosophy of Viktor Frankl, who believed that it was best to focus on what is left rather than what is lost whenever possible. Of course, this is easier said than done. Dr. Panter-Brick, your comments are also related to optimism or the belief that things will work out.

### Dr. Bonanno: Multi-determinate predictors

We seem to have moved from the minute to the broad. I'm going to take us back to the minute, to the data. It's interesting that you brought up Viktor Frankl because he wrote decades ago. His ideas are moving and important but in my opinion those ideas take us only so far. I've argued recently that the word resilience is almost useless as a single word and that it really only makes sense if we qualify it. For example, the type of process that Dr. Yehuda brought up is very different than the kind of process that I've been studying and that Dr. Masten and Dr. Panter-Brick were talking about. We introduced the phrases “minimal-impact resilience” and “emergent resilience” as two forms of resilience (Bonanno & Diminich, 2013) but I think there are potentially other forms. We are also able to detect these patterns in animals exposed to threat (Galatzer-Levy, Bonanno, Bush, & LeDoux, 2013). In most of our work, we have focused on minimal-impact resilience in response to acute adversities. We have thus far determined that there are five basic categories of factors that predict this kind of minimal-impact trajectory (Bonanno et al., 2011). First there are economic resources, as Dr. Stevan Hobfall has been talking about for years (see Hobfoll et al., 2007). Resources are incredibly important, although we don't talk about them much when we talk about resilience because they're basic, not quite as sexy, and building resources costs a lot of money. Then there are social resources. There is personality, which is something we all love to think about, and genetic factors. But personality and genes are just two of the many pieces of the puzzle and they are actually small pieces. If we measure many different predictors, we find that no one predictor accounts for much variance. What I mean is that no single demographic, personality or biological factor has been shown to predict or enhance resilience by more than a small degree. My approach is to study a specific kind of resilience, minimal-impact resilience, as well as a specific set of factors that each may on their own contribute a relatively small piece of the puzzle. From there we can work our way to a broader picture. So I'm really advocating bringing it back down to a more focused empirical perspective.

### Dr. Yehuda: Trauma does not only yield pathology

Please permit me to comment on that, because I don't think that the goal is to come up with one definition for resilience. I think that it is fantastic that different people are looking at the phenomenon of resilience from different contexts. It is important for anyone that does a specific piece of research to let everyone know what their question is and what was studied and what the specific outcome variables were. We should absolutely not restrict the field by tethering it to one person's conception of resilience. But I wanted to make another point as I am listening to all of these wonderful comments. I think it is important to reflect on the fact that here we are at an international meeting about traumatic stress and we are having a plenary on the topic of resilience, not trauma. This is an extraordinary development and represents the desire of the field to not be hijacked by pathological symptoms or negative effects of trauma. So it doesn't really matter if we have different definitions of resilience. It matters that we continue to have a conversation about resilience because the meta-message is that the experience of trauma does not only yield pathology.

### Dr. Masten: Contextual and cultural considerations in defining adaptive function

I do think it's important, as I think Dr. Bonanno was highlighting, to distinguish between concepts and models and how we approach our empirical work and it's critical that we define our criteria very, very carefully in our empirical work. Each of us does research in different areas, although some of our research probably overlaps. It is extremely important to very carefully define the criteria of adaptive function and adversity for your studies, the levels of analysis and processes that you are attempting to measure.

In regard to this question, what have we learned about what makes a difference? There is a huge literature now on the topic of resilience in children and youth (e.g., Cicchetti, 2010, 2013; Masten, 2011, 2014a, 2014b; Panter-Brick & Leckman, 2013; Ungar, 2008, 2012; Ungar, Ghazinour & Richter, 2013). There clearly are some particular protective and resilience-enhancing factors that are implicated over and over again as important across a wide variety of circumstances, such as children having a protective parent on the scene who is functioning pretty well and protecting the child. But there is also a great deal of diversity in this literature as well. If you take a specific context and look at particular criteria for defining adaptive function you will invariably get a somewhat different understanding of protective factors that matter, and this is very striking when you look at the global research on resilience in children and adolescents. You may think you have some important process thought through carefully and then something provocative will come up. For example, I am very fond of the notion that agency, along with the pleasure and perceived mastery that goes along with that adaptive system, is a powerful protective factor in human development (Masten, 2014b). However, research on youth who become involved in political violence in Middle-Eastern conflict-prone areas indicates that they become engaged at least partly because it gives them a sense of mastery and involvement (Barber, 2009). These findings are provocative and they make you think—okay, wait a minute here—always, always, resilience has context (Wright & Masten, in press). We have to be very precise about what contexts we are studying.

Dr. Panter-Brick and I were involved in a forum recently that posed the question, “What can we do in early childhood to promote peace?” (Leckman, Panter-Brick, & Salah, in press). That was a tough question. It could refer to peace in a school, peace in a community or global peace but it was a very provocative question. When you are confronted by the empirical evidence from very different circumstances, it sharpens your thinking. It is important to know—whether it is for young people in Afghanistan or other young people around the world—what facilitates resilience? I think that we are moving toward a more personalized version of resilience that is embedded in context. We can learn about general principles of resilience but the reality is that people differ and for some individuals, different protective factors may be important for specific outcomes in specific contexts.

### Dr. Southwick: Understanding resilience from a multidisciplinary perspective

This is a great challenge: how to understand resilience from a multidisciplinary stand point. How do you measure resilience from a multidisciplinary stand point? Where do you begin? Where have you begun Dr. Masten?

### Dr. Masten: Interdependence of systems

I think that there are global challenges where people had to sit down and think about this issue, for example to think about how to prepare a population for disaster. In Minnesota we don't prepare for hurricanes, but we prepare for other kinds of disasters that could occur there. In thinking about disaster planning, you think about the systems involved in human life and adaptation in a given context. The lives of children are embedded in families and schools, as well as communities and cultures. What you do is get teams of people together that represent different areas of relevant expertise, different sciences and intervention realms, having to do with schools and families and community and state and emergency response systems. Then you sit down and try and figure out what can the community and emergency response system do to be more supportive to family resilience (Masten & Monn, in press) because that's going to be important to children and so forth. How do we prepare first responders so they can operate with an awareness of all the different systems that are involved in emergency response, including schools, families, and children? One of the reasons it is taking so long to recover from Hurricane Katrina, and now from Superstorm Sandy, is because so many systems that are interdependent collapsed all at the same time or were damaged. It takes a while to build them up.

People are learning to get together and put together plans and solutions focused on integrated responses to a particular kind of problem, such as a flu pandemic, a hurricane, or a terrorist attack, etc. They are trying to think through the key systems involved and what we know about fostering and supporting resilience in those systems because if you ever go through a major disaster you are deeply imprinted with the realization that your own resilience is highly interdependent on many other layers of systems and how they're operating.

There is an old saying that “all disasters are local.” This idea comes from the reality that disasters can knock down many systems at once, including communications systems, leaving people to rely on local systems in the immediate environment. But as the recovery goes forward, major systems, including communications, are being restored and rebuilt. Planning for disasters and recovery needs to consider the adaptation of interconnected systems, the needs of children and families, and how to support recovery at different stages of rebuilding. There is growing traction on these issues in disaster planning, both in the United States and around the world (Masten & Narayan, 2012).

### Dr. Southwick: Technologies and resilience

When we think of resilience, we often focus on the individual but in fact we need to consider embedded systems. How about technology and resilience? How are new technologies and research in fields such as genetics, epigenetics and brain imaging informing the science of resilience?

### Dr. Yehuda: Biology and the study of resilience

It is not yet clear exactly if or how the science or biology of resilience is going to impact the way we deal with trauma in the context of systems. I think there is a real opportunity for science to inform us about the more narrow question of recovery from certain kinds of consequences that are maladaptive. If we understand the kind of biological underpinning of symptoms, then we may be able to have effective interventions for those who we know are going into harm's way, or we may be able to identify those people more rapidly and then build resilience programs on an individual level. But scientific advances hold great promise for helping us in very important ways. For example, if there was a specific imperative (e.g., can a soldier who is in the field be safely returned to the combat theatre?), there may be a real place for biology to help answer that question in the future – uniquely – as a novel assessment mechanism. Or if you think about the biological measures predicting recovery or treatment, there might be an ability to use technologies and research and genetics or epigenetics or molecular biology to match people to the interventions that are going to be most likely to help them achieve success. Those would be very important contributions to individuals and society.

### Dr. Bonanno: Technology and social capital

I have a different twist on the question. The role of technology in how people deal with adversities became very apparent during Super Storm Sandy in New York where I live. There was a great example of the use of texting. The local power company was trying to tell people what was happening but the power station blew out and Lower Manhattan was in darkness. People in the outer lying areas had lost power and that's a big issue when you're trying to cope with disaster. So people were texting each other right away with important information and updates. This got me very interested in the crucial importance of social capital. Then, not long after, I was asked to speak with the principals of the schools that were knocked out by Super Storm Sandy. I presented my research and they told stories of their experiences. Their stories were all about social capital: they were all about having lines of communication with other people; about knowing where the resources where. So this kind of phenomenon seems very important. There's a lot of theory about social capital but it's very poorly studied in relation to psychological functioning. We did manage to collect data on this in New Jersey, after the storm, and we are in the process of linking this data with prospective data that had been collected previously by Rachel Pruchno. I think these kinds of technological applications may be crucial and can be fostered.

### Dr. Yehuda: Using change in biology to understand trajectories of resilience

I was hoping to hear from Dr. Bonanno about whether any biological changes have been observed in association with the trajectories of resilience that he has studied. Of course, technology is always going to improve our lives, but those of us in the neuroscience space wonder how this work helps shape our conclusions about these psychological constructs because, to date, our policies are almost exclusively driven by sociology or psychology. If neuroscience confirms the trajectories, that would be important information, as would be a disconfirmation by biological data.

### Dr. Bonanno: Combining analytic and biological methods

I'm not a neuroscientist but I'm absolutely fascinated by the stress response equipment that we have; it's not only genetic, or the amygdala, or the hippocampus. It's the whole integrated system and it works amazingly well. So when something really life threatening, some fear-inducing event says, “you are in big trouble, do something,” that's when initially the catecholamines kick in and we prepare ourselves for flight or fight response. A little bit later the hypothalamic pituitary adrenal axis and the cortisol system is activated and the way this works absolutely fascinates me. We have an immediate response so we can react right away—a little bit like crying for help—and then we have a longer term response related to cortisol and peptides and other neuroendocrine mechanisms that only come on line when we're dealing with an enduring stressor. When I first learned about this process, I wondered why we would send a neuroendocrine response via the blood, from the brain way down to the adrenal glands. Why did nature evolve such a circuitous path? Part of the answer is precisely because it's slower. When the slower response finally does come online, we're really dealing with a more powerful response, almost an alternate state of consciousness. Coming late to this as a kind of novice, I find this amazing. But then it raises this great question of why does this not work for everyone? Dr. Yehuda has done some really important work in this area, along with Drs. Southwick and Charney. This to me is really where the money is in terms of figuring out, at least internally, the stress system. But what I think needs to happen is this work needs to be done in concert with research on different outcome patterns, trajectory analyses. This is, of course, very easy to say and very hard to do. In my lab we have been mapping outcome trajectories using sophisticated latent modeling procedures. We are also attempting to integrate this approach with the use of experimental procedures. It's complicated because latent modeling requires large samples, but experimental, and of course biological, procedures have to test people one at a time. The real work that needs to be done hasn't quite been done yet, but I think it's enormously important. I think doing this kind of work in a context of trajectory analyses would really do a lot to distinguish why some people are resilient and some are not.

### Dr. Panter-Brick: Biomarkers of resilience

I want to briefly talk about what we call the biomarkers of resilience. These include measures of blood pressure, stress hormones, immune function, and gene methylation. We can use these biomarkers to help us connect the dots between the neurobiology and physiology of resilience and the culture of resilience. Let's say we want to know the extent to which an intervention to reduce stress really works—perhaps an intervention such as “mindful meditation” or a “psychosocial” intervention to treat or alleviate traumatic stress symptoms. One powerful use of biomarkers would be to measure physiological stress before and after such an intervention. I am advocating this approach, because I do think that using biomarkers for program evaluation is a growth area for research in the future. This is not just because we want to willy-nilly use biomarkers to measure the signatures of adversity on the human body, but because we think that, once we understand resilience, we should be savvy in measuring indicators of change in resilience-building interventions over time. Biomarkers offer us an evaluation tool other than self-reported data on feelings and behaviors. They help us understand the mechanisms through which risk and resilience leave epigenetic and physiological signatures on the body, which have developmental implications for young children and long-term health implications for adults.

### Dr. Masten: Leveraging new tools and technology in science

Since I began graduate school, the transformation in tools and technology available to study resilience is staggering. Back then, we assumed there was a neurobiology of resilience but measures were unavailable or were impractical. At that time it was very expensive or very difficult to study the neurobiology of resilience. Now investigators are doing all kinds of fascinating work—watching the brain in action during adaptation or measuring epigenetic change, not only as an indicator of adaptive function, but also as a moderator of response to interventions. We also now have the capabilities through widespread use of the Internet to collaborate with people around the world and upload and feedback data from the field. The measures we are capable of getting out there in the field and practically in the middle of nowhere are having a huge impact on the science of studying adaptation. Another important area of advancing methodology in resilience is statistics. We had all this lovely theory about trajectories and patterns of resilience but now we are able to get repeated measures and use growth analyses either to study the patterns of change over time or to extract and test our ideas about pathways. Are there real life trajectories like we hypothesized a few decades ago? Now we have capabilities and tools at our finger tips or through collaborations that are transforming the way we think about resilience and how it works.

### Dr. Southwick: Enhancing resilience

Can the capacity for resilience be enhanced or taught? I think we probably would all agree “yes,” but from your perspective, what are the most effective ways to enhance resilience. Please address the question from your area of research.

### Dr. Bonanno: Regulatory flexibility

I want to point out that we have to be very careful here because resilience is common. If we think about something prophylactically, we have to make sure that we are not undermining people's natural resilience. For example, if we look at the literature on human factors, we see that interventions like bicycle helmets, or seat belts, which make people safer actually tend to increase accidents. That's because people feel safer so they become less cautious. There are a lot of different factors that might make people resilient, and if we're talking about enhancing these factors we have to target which factors are most feasible (for a review, see Bonanno et al., 2011). I'm very interested in a concept we are calling “regulatory flexibility” (Bonanno & Burton, 2013). We are focusing on “flexibility” in my lab (e.g., Bonanno, Papa, Lalande, Westphal, & Coifman, 2004) because it seems to me to be “learnable.” It's the basic idea that how you cope or deal with a situation depends completely on the situation, so it applies to some of the global concepts that Dr. Panter-Brick and Dr. Masten have brought up. We have argued that there are three key components to flexibility: (1) How we read the situation, or context sensitivity; (2) a repertoire of behaviors, and (3) the ability to regroup using corrective feedback (Bonanno & Burton, 2013). The underlying idea is that there isn't a right or perfect way to cope. It all depends on the situation—that idea alone is news to some people. I am often asked by the media to comment on major events, such as the Boston Marathon bombing. The questions are often about what people should do, what is the best way to cope. And I often find myself saying it depends on who they are, what happened to them and what the situation is.

### Dr. Panter-Brick: Dignity and achieving a “good enough life”

I think that the most important and effective way to approach resilience is to start with listening to what people have to say about their everyday lives. I want to understand what goals are important, and identify what people are already doing for themselves to reach them. Resilience is about achieving a “good enough life”—there is a normative dimension to realizing your own goals that is very important (Panter-Brick & Eggerman, 2012). In that sense, resilience is doing more than just “functioning well” or “better-than-expected.” It is about “making sense” of the moral aspects of your life. So the first thing I would do to identify resilience is to talk with people and listen to what their goals are. I'll come back to the example of Afghanistan, where families tell us they suffer the drip, drip, drip of multiple everyday stressors, engendered by war, poverty, social inequality, family quarrels, and community conflict. But Afghans will also tell us that what matters most to life is sustaining a sense of hope and dignity. Indeed, Dr. Ashraf Ghani, former Finance Minister and current President, emphasized that human dignity should be front and center of plans for social and economic development. He told the United Nations Development Programme (UNDP) that investments in Afghanistan might not allow the country to become a middle-income country, but would allow it to move from ‘abject poverty’ to ‘poverty with dignity’.

Here we see that effort to sustain dignity, rather than simply to alleviate misery, is the key to a hopeful future. If human dignity is the most important goal here, then a mental health intervention to alleviate suffering after exposure to acute and/or chronic stressors could actually include the provision of key social, economic, and political resources providing families with housing, jobs, education, and secure neighborhoods. There are a lot of things we can do in terms of social justice to bring about greater equity in society. I think that interventions targeted at readiness for jobs and education, targeted at alleviating violence and human insecurity, or targeted at social justice to enhance fairness in access to resources are among the most effective ways to enhance resilience. I'm not being fuzzy here, I'm being really serious—remember, I don't endorse approaches that are hazy, light, or lame! Taking the perspective of any parent whose children are at risk, I am here emphasizing that interventions that take only a piecemeal or short-term action to boost physical and mental health do not necessarily resonate with my cultural goals. What may matter more to me is that my children will get a fair deal in society and have a decent life, so that human dignity is not incessantly eroded. So listen to my cultural goals, because those are the ones that matter for my family to survive and thrive.

### Dr. Masten: Promoting healthy development and supporting adaptive systems

For me this will depend a lot on what your timing is. If you are trying to enhance resilience from a developmental point of view the best thing you can do is to promote healthy development, to make sure that the brain is developing in healthy ways, that the family caregiving system is working well, and so forth, so that you end up with populations of people who have developed their capacity for adaptation. Our species has great potential for adaptive capacity if we provide a healthy context for development. I'm extremely concerned in this country that we are allowing so many children to be harmed by toxic levels of stress exposure that affects their capacity to adapt before they barely get off the ground. I study children in homeless families in the Twin Cities and it is frightening to see how much damage can be done before you even get to kindergarten by having overwhelming levels of trauma and adversity day after day.

I would support key natural protective systems for child development, especially families, so they can provide what their children need. As a nation, we also need to support communities so that they can provide families with the resources they need – whether that is economic resources, emergency supplies, water, or whatever is required (Norris, Steven, Pfefferbaum, Wyche, & Pfefferbaum, 2008; Norris et al., 2009). I really think it's important to support these natural adaptive systems for children and families—the engines that provide so much of the power for resilience (Becvar, 2013; Masten, 2014b; Masten & Monn, in press).

### Dr. Yehuda: Enhancing resilience before the trauma

Ideally, we want to enhance resilience before trauma occurs by practicing how we would respond to a trauma. We don't do that in our culture. We like to live our lives with the idea that nothing bad will happen and everything is going to be all right. And so that is the message that we give our children: that everything is going to be all right. And that's what we tell our selves—everything is going to be all right. Perhaps it would be more prudent to prepare for adversity. According to statistics, we know that the probability of trauma occurring is high, so we don't have to wonder if trauma exposure will occur, but when is it going to happen? And we must prepare early on. What are the ways—on an individual level that one can use resources to cope with adversities so that for starters, exposure is not such a shock. After trauma occurs the way to enhance resilience is to find the places where there are strengths. Maybe there is natural resilience. Maybe it is necessary to have a really good infrastructure to help those who are less naturally resilient. Maybe it is important to have a good community. Different people are going to need different things to actualize their resilience. But we have to look for the thing that is present for that individual and go with it so that there is at least one strong foundation on which to build more resilience. I agree with what has been said, but I think that a culture that expects to have to deal with adversity will deal with it better, and we have not spoken at all about preparation, which may be an important key.

## Discussion

In this International Society for Traumatic Stress Studies presentation, an interdisciplinary group of experts tackled some of the most pressing current questions in the field of resilience research, generating a lively discussion on need for definitions of resilience, the most important determinants of resilience, new technologies that may inform the science of resilience, and lastly, the most effective ways to enhance resilience.

### How do we define resilience?

Proposed definitions included a stable trajectory of healthy functioning after a highly adverse event; a conscious effort to move forward in an insightful and integrated positive manner as a result of lessons learned from an adverse experience; the capacity of a dynamic system to adapt successfully to disturbances that threaten the viability, function, and development of that system; and a process to harness resources in order to sustain well-being. A number of these definitions bring into question the notion that resilience is characterized by the absence of functional impairment or psychopathology following highly adverse events. For example, should we classify a trauma survivor as resilient if that person develops chronic symptoms of PTSD but also functions at a high level, because they have succeeded in seeking out and using ample personal, material and social resources?

All panelists stressed the importance of continued research directed toward establishing empirically driven operational definitions of resilience, recognizing that resilience is a complex construct that may have specific meaning for a particular individual, family, organization, society and culture; that individuals may be more resilient in some domains of their life than others, and during some phases of their life compared with other phases; and that there are likely numerous types of resilience (e.g., acute resilience; emergent resilience) that depend on context (e.g., resilience for a traumatized Cambodian refugee may be different than resilience for an American who lives through a hurricane, or than an individual suffering with chronic schizophrenia). On the one hand, the goal may not be to agree on one definition of resilience, but rather to carefully define various types of resilience depending on context. On the other hand, in order to establish a single broader, but nevertheless useful, definition of resilience, it will be essential to collaborate with experts who study engineering, ecological, biological, individual, family, organizational and cultural resilience.

### What are the most important determinants or drivers of resilience?

Panelists discussed the need to approach our understanding of resilience and its determinants from multiple levels of analysis, including genetic, epigenetic, developmental, demographic, cultural, economic and social. In research to date, specific determinants generally serve as relatively weak predictors of resilience by themselves and explain a relatively small piece of the puzzle. An exception may be childhood protective factors that are routinely identified as being important for developing resilience. These include a healthy attachment relationship and good caregiving, emotion regulation skills, self-awareness and the capacity to visualize the future, and a mastery motivation system that drives the individual to learn, grow and adapt to their environment.

Determinants of resilience may also differ depending on context and specific challenges. For example, some of the determinants of resilience that are relevant for a firefighter in the United States may differ from those that are relevant for a mother living in an impoverished country. While it is useful for researchers to identify general principles related to resilience, it is also important to recognize that successful determinants may vary from one person to the next based on multiple factors such as personality, specific challenges, resources available, and environmental context. In addition, there is evidence suggesting that resilience is associated with the ability to employ a variety of coping strategies in a flexible manner depending on the specific challenge, and then to use corrective feedback to adjust those strategies. Further, the determinants of resilience may vary depending on the age and maturity of the individual. For example, having parents that are highly protective may foster resilience during infancy and early childhood but not during later childhood and adolescence.

### How are new technologies informing the science of resilience?

Recent and rapid advances in neurobiology (e.g., brain imaging, genetics, epigenetics) hold great promise for elucidating mechanisms of trauma-related symptom development as well as mechanisms of successful adaptation to, and recovery from trauma. A more complete understanding of underlying neurobiology may make it possible to identify pre-existing strengths and vulnerabilities; to distinguish between and predict trajectories of symptom development and/or resilience following a trauma; to develop more scientifically informed, targeted and individualized strategies for treating trauma-related symptoms as well as for building specific skills designed to foster resilience.

Other areas where technological advances have and will continue to rapidly advance the field include novel statistical approaches to data analysis and widespread use of methods, such as the Internet, to share knowledge and to connect research participants with resilience-focused research scientists around the world.

### What are the most effective ways to enhance resilience?

In order to develop effective interventions to enhance resilience, it is critical to understand that humans are embedded in families, families in organizations and communities, and communities in societies and cultures. Interventions targeted at any one of these levels will impact functioning at other levels. Sometimes the most effective strategy to enhance resilience at a specific level may involve intervening on a different level. For example, to enhance resilience in a young child it may be more effective to provide schools and parents with needed resources (e.g., healthy meals; education on how to raise children) than to intervene at the level of the individual child. Similarly, communities may enhance individual resilience by providing job training and placement for those who are unemployed. In other words, resilience in the individual is highly dependent on multiple layers of society.

It is also important to understand that determinants of resilience in one community may differ from those in another community (e.g., rural Afghanistan vs. Manhattan), and that some skills needed to successfully deal with one stressor/trauma may differ from those needed to cope with a separate traumatic situation (e.g., terrorist attack vs. cancer diagnosis). For example, instilling a sense of hope, dignity and coherence may be critically important for fostering resilience in a war torn and impoverished community but not in a stable and resource rich community. To develop effective resilience-enhancing interventions that are informed by an understanding of these complexities, experts from a broad range of disciplines will need to work together and listen carefully to one another as well as to those who are actually facing trauma.

Regarding children, perhaps the most effective way to enhance resilience is to provide a safe, stable and loving environment that allows the child's natural protective systems to emerge, and to foster healthy brain, cognitive, emotional and physical development. In order to improve the odds for healthy development and resilience, it may be necessary to provide a variety of resources to families, schools and communities.

Interventions to enhance resilience can be administered before, during or after stressful/traumatic situations. Some interventions may be more effective at one time point than another. Ideally, interventions/training will occur prior to stressful events so that the individual is better prepared to deal with adversity.

Humans are endowed with great potential to weather adversity and to change or adapt when necessary, but they need basic social and material resources to do so. One of the most important ways to foster resilience is to promote healthy family and community environments that allow the individual's natural protective systems to develop and operate effectively.
